# Singing streams: Describing freshwater soundscapes with the help of acoustic indices

**DOI:** 10.1002/ece3.6251

**Published:** 2020-04-16

**Authors:** Emilia Decker, Brett Parker, Simon Linke, Samantha Capon, Fran Sheldon

**Affiliations:** ^1^ Australian Rivers Institute Griffith University Nathan Queensland Australia

**Keywords:** ecoacoustics, freshwater acoustic, freshwater streams, indices

## Abstract

1. Understanding soundscapes, that is, the totality of sounds within a location, helps to assess nature in a more holistic way, providing a novel approach to investigating ecosystems. To date, very few studies have investigated freshwater soundscapes in their entirety and none across a broad spatial scale.
2. In this study, we recorded 12 freshwater streams in South East Queensland continuously for three days and calculated three acoustic indices for each minute in each stream. We then used principal component analysis of summary statistics for all three acoustic indices to investigate acoustic properties of each stream and spatial variation in their soundscapes.
3. All streams had a unique soundscape with most exhibiting diurnal variation in acoustic patterns. Across these sites, we identified five distinct groups with similar acoustic characteristics. We found that we could use summary statistics of AIs to describe daytimes across streams as well. Most difference in stream soundscapes was observed during the daytime with significant variation in soundscapes both between hours and among sites.
4. *Synthesis and Application. *We demonstrate how to characterize stream soundscapes by using simple summary statistics of complex acoustic indices. This technique allows simple and rapid investigation of streams with similar acoustic properties and the capacity to characterize them in a holistic and universal way. While we developed this technique for freshwater streams, it is also applicable to terrestrial and marine soundscapes.

## Graphical abstract

Understanding soundscapes helps to assess nature in a holistic way and provides a novel approach to investigate ecosystems. In unprecedented spatial and temporal resolution, we recorded 12 freshwater streams in south‐east Queensland for multiple days, calculated three acoustic indices and used a principal component analysis to investigate acoustic properties of each stream. While all streams had a unique soundscape, we could also identified five distinct groups with similar acoustic characteristics across all sites, showing our technique allows a rapid investigation of stream soundscape.

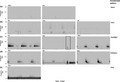

## 1 INTRODUCTION

There is growing recognition that ecosystems can be described based on the complexity of the sounds they produce (Farina, 2014). Soundscapes can be defined as “the totality of all sounds within a location with an emphasis on the relationship between an individual's, or society's, perception of, understanding and interaction with the sonic environment” (Payne, Davies, & Adams, 2009). Soundscapes provide a unique insight into both the biotic and abiotic components of ecosystems (Farina, 2014) including terrestrial (Farina & Fuller, 2017), marine (Putland, Constantine, & Radford, 2017), and freshwater (Linke, Decker, Gifford, & Desjonquères, 2020) systems. Within ecosystems they have been described for many individual biotic components including mammals, birds (e.g., Elemans et al., 2015), amphibians (e.g. Clulow, Mahony, Elliott, Humfeld, & Gerhardt, 2017), invertebrates (e.g., Sweger & Uetz, 2016), reptiles (e.g., Young, 2003), and fish (e.g., Rountree & Juanes, 2018). Despite this broad range of soundscape description, there have been few attempts to summarize soundscape complexity across space and time and thereby provide further insights into aspects of ecosystem function.

Ecosystems are typically described in relation to their species’ complexes, functions and physical/chemical processes (Ulgiati & Brown, 2009) which collectively tend to vary with respect to three major dimensions of biocomplexity, that is, spatial heterogeneity, organizational connectivity, and temporal contingencies (Cadenasso, Pickett, & Grove, 2006). These ecological dimensions can be summarized by a range of indices and metrics such as taxonomic richness, tolerance‐based biological indices, functional redundancy, and response diversity (Soria et al., 2019). Indices can be used to detect anthropogenic impact in different ecosystems (Mouillot, Graham, Villéger, Mason, & Bellwood, 2013; Soria et al., 2019) and monitor both natural and disturbed systems (Belmar et al., 2019; Bruno, Gutiérrez‐Cánovas, Velasco, & Sánchez‐Fernández, 2016). Further indices, such as functional diversity (Laliberte & Legendre, 2010), biodiversity (Izsák & Papp, 2000), and species richness (Heltshe & Forrester, 1983), are also useful for describing complex ecological contexts in a simple and universal way.

River habitats have traditionally been characterized according to physical, chemical, and ecological parameters (Leopold & Maddock, 1953; Montogomery & Buffington, 1997; Wohl & Merritt, 2008); however, very little is known about their acoustic character. To date, freshwater acoustic research has mainly focused on describing the biological sounds produced by specific soniferous species like fish (Anderson, Rountree, & Juanes, 2008; Montie, Vega, & Powell, 2015) and insects (Desjonquères et al., 2015) as well as nonbiological sounds produced by physical processes such as sediment transport (Tonolla, Lorang, Heutschi, Gotschalk, & Tockner, 2011). Some human‐generated sounds, such as the sounds emitted from boats, have also been investigated (Amoser, Wysocki, & Ladich, 2004; Wysocki, Dittami, & Ladich, 2006). In contrast, very few studies have explored the freshwater soundscape as a whole (e.g., Desjonquères et al., 2015; Linke et al., 2020).

Rivers are longitudinal systems, connected from their headwaters to lower reaches by the downhill movement of water (River Continuum Concept: Vannote, Minshall, Cummins, Sedell, & Cushing, 1980). Along this continuum, they vary spatially and temporally over many scales in terms of flow and sediment regimes (Ward, Tockner, Uehlinger, & Malard, 2001) and likely the same is true for their soundscapes. In their lowland reaches, rivers are connected laterally to their floodplains (Junk, Bayley, & Sparks, 1989). Hydrological connectivity between floodplain habitats can influence the range of recorded biotic sounds, with more hydrologically connected sites sharing similar acoustic soundscapes and macroinvertebrate communities (Desjonquères, Rybak, Castella, Llusia, & Sueur, 2018). Factors contributing to these spatial patterns in sound, however, remain poorly understood.

The most fundamental physical attribute that varies across freshwater systems is the presence, or absence, of flowing water. Lentic habitats, for instance, generally have lower sound levels compared with lotic habitats (Wysocki, Amoser, & Ladich, 2007). Furthermore, the relative roughness of habitats (i.e. relative submergence) at a site predominantly affects middle sound frequencies (63 Hz–1 kHz) while streambed sediment transport can increase sound pressure level (SPL: the effective sound pressure relative to a reference value [Madsen, 2005]) in the high frequencies (2–16 kHz) (Tonolla, Acuña, Lorang, Heutschi, & Tockner, 2010). Across a range of studies, SPL across the entire soundscape tends to increase in relation to flow level and flow velocity. However, rivers with lower physical heterogeneity and limited local sediment supply and transport tend to exhibit the most homogeneous soundscapes (Tonolla et al., 2011). Differences in soundscapes among freshwater habitats are therefore driven not only by their biotic communities, but also their physical attributes.

In addition to spatial variation, freshwater soundscapes also exhibit multiple levels of temporal variation. Fish, aquatic insects, and hydraulic sounds often occur during specific times of day and, depending on the freshwater system, dusk and dawn periods can have high sonic activity (Linke, Decker, Gifford, & Desjonquères, 2020) or none at all (Gottesman et al., 2020). While most sounds exhibit a diurnal pattern, some might occur only rarely or be more frequent after rainfall (Gottesman et al., 2020). Seasonal sound patterns are less explored in freshwater systems, though fish have been found to have “species specific” seasonal patterns with fish sound production often beginning in spring, continuing into autumn and not occurring during winter (Montie et al., 2015).

One way to simplify acoustic data is to use acoustic indices, that is, summary metrics analogous to ecological indices that can be used to expose underlying patterns associated with certain characteristics of soundscapes such as loudness and complexity. These indices characterize soundscapes by summarizing either the whole soundscape or a specific frequency range (Sueur, Farina, Gasc, Pieretti, & Pavoine, 2014) and allow for overall diversity comparison between different sites (Gasc, Sueur, Jiguet, et al., 2013). This is especially useful in freshwater systems where other noninvasive methods like visual detection are limited by vegetation and turbidity. In freshwater systems, acoustic indices have been applied to detect daily acoustic patterns of fish, aquatic insects, and streamflow (Linke et al., 2020) as well as seasonal acoustic dynamics across the whole soundscape (Gottesman et al., 2020). Acoustic indices have been used to measure acoustic diversity (Desjonquères et al., 2015) and acoustic richness (Gottesman et al., 2020). Further, acoustic indices have been employed to distinguish species and describe acoustic features such as call rate and call amplitude (Indraswari et al., 2018). Though several freshwater studies have used acoustic indices in recent years (e.g., Desjonquères et al., 2015; Gottesman et al., 2020; Linke et al., 2020), no study to date has investigated the whole soundscape of more than six sites.

Here, we use summary statistics of acoustic indices to explore the spatial and temporal diversity of freshwater stream soundscapes across 12 sites in South East Queensland, Australia. Our aim was to characterize the spatial patterns in soundscapes across multiple stream systems, to classify streams according to their soundscape and explore the potential use of acoustic indices to describe stream soundscapes. We focussed our study on the soundscapes of lowland streams and hypothesized that soundscapes will be unique to particular stream types and acoustic indices will be able to adequately describe the soundscape patterns. We recorded 12 lowland streams for three days and characterized the soundscapes on a 24 hr and daytime scale (i.e., grouping hours into day, night, and twilight hours) using three acoustic indices, each of which describes different aspects of the soundscape: (1) *M* (Median of amplitude envelope); (2) H (Acoustic entropy index); and (3) ACI (Acoustic complex index). We then used summary statistics of these acoustic indices in a principal component analysis (PCA) to evaluate soundscape similarity between sites and times of day.

## 2 METHODS

### 2.1 Study area

This study was conducted in South East Queensland (SEQ) on the subtropical, eastern coast of Australia. The region comprises 15 major catchments with a combined area of almost 23,000 km^2^. SEQ is the fastest‐growing region in Australia with an estimated projected growth from 3.5 million to 5.3 million people over the next 25 years (Department of Infrastructure Local Government & Planning, 2017). The receiving waters of Moreton Bay and its estuaries have very high conservation value and support fisheries and tourism, while the western catchments are the region's primary water supply (Zhou, Li, Tao Shen, Kitsuregawa, & Zhang, 2006; Bunn et al., 2010). Since the late 1800s, European settlement has left a significant ecological footprint in the region, resulting in substantially altered catchment hydrology and numerous environmental concerns including significant declines in water quality and biodiversity loss (Bunn et al., 2010; Zhou et al., 2006). For more information on the SEQ region see Bunn et al. (2010).

Sites for this study were selected from a pool of locations used in a large‐scale monitoring program that has been conducted across SEQ since the 1990s (Bunn et al., 2010; Healthy Waterways, 2018). The Healthy Land and Water monitoring program provided a comprehensive assessment of river health and the response of aquatic ecosystems to human activities (e.g., catchment alterations) for each of SEQ’s major catchments, river estuaries, and Moreton Bay zones(Bunn et al., 2010; Sheldon et al., 2012). We used the Healthy Land and Water monitoring data to identify 12 suitable lowland creeks (stream order < 4) (Table 1) and analyzed their soundscape.

**TABLE 1:** Streams with site code and HWL name

Site code	Site name	Waterway	Longitude	Latitude	Recorded
Emu	Grieves Road, Colinton	Emu Creek	152.2928	−26.96295	7–10.4.2018
Bua	Rocky Gully Road, Coominya	Buaraba Creek	152.4336	−27.3975	11–14.4.2018
Lai	Railway Bridge, Gordon Street, Forest Hill	Laidley Creek	152.377	−27.60235	11–14.4.2018
San	Wivenhoe‐Somerset Road, Crossdale	Sandy Creek	152.5554	−27.2246	11–14.4.2018
Bur	Boonah Rathdowney Road, Maroon	Burnett Creek	152.6803	−28.1701	14–17.4.2018
Cai	Cainbable Creek Road, Kerry	Cainbable Creek	153.0793	−28.0967	14–17.4.2018
Wa1	Villis Bridge, Niebling Road, Tarome	Warrill Creek	152.4784	−27.9886	14–17.4.2018
Wa2	Kalbar Connection Road, Kalbar	Warrill Creek	152.6003	−27.93221	14–17.4.2018
Del	Dewhurst Road, Mount Delaney	Delaney Creek	152.715	−27.00615	18–21.4.2018
Kan	Kangaroo Creek Road, Moore	Kangaroo Creek	152.3809	−26.8806	18–21.4.2018
Mon	Monsildale Creek Road, Linville	Monsildale Creek	152.2814	−26.78268	18–21.4.2018
She	Crossing No 2, Kilcoy ‐ Murgon Road, Kilcoy	Sheepstation Creek	152.5246	−26.8669	18–21.4.2018

### 2.2 Recording technique

We used four Aquarian Audio H2a Hydrophones (Sensitivity: −180 dB re: 1V/µPa) and four Zoom H6 Handy Recorders (44.1 kHz/16bit, maximum gain with a −20dB pad) at all the sites. A recorder and hydrophone were placed at each site for 72 hr with the hydrophone placed in the middle of the stream. Data were saved on *SD* cards before transferred to an external hard drive. Recording took place from 7th to the 21st of April 2018. (Table 1). During this time, sunrise was around 06:00 and sunset around 17:30. Visualization of recordings was accomplished by subsampling recordings for periods of 1 s at intervals of every 10 min (*sensu *Linke et al. 2020) and using the multimedia software ffmpeg (Hann windows of 1,024 samples with 80% overlap, FFmpeg, 2019) to produce spectrograms.

### 2.3 Data analysis

#### 2.3.1 Variation in acoustic soundscapes between sites

To find acoustic properties that best described variation in recorded soundscapes between sites, we calculated three acoustic indices (AIs: Table 2) for every minute of every hour of recording from each site using the R packages Seewave (Sueur, Aubin, & Simonis, 2008) and Soundecology (Villanueva‐Rivera & Pijanowski, 2016). Summary statistics for each hour at each site (i.e., minimum, maximum, median, mean, standard error, standard deviation, 95% confidence interval, variance, coefficient of variation, and interquartile range) were then calculated separately for each AI using the R package pastecs (Grosjean, 2018). We used a combination of all three acoustic indices, as the aim was to characterize the soundscapes in a holistic way rather than look at which AI separates the soundscapes of sites better.

**TABLE 2:** Acoustic indices used in this study

Full name	Abbreviation	Principle	References
Median of amplitude envelope	M	The median of the amplitude envelope, which is an indicator of overall sonic activity	Depraetere et al. (2012)
Acoustic Entropy Index	H	A measure of complexity in both time and frequency	Sueur, Pavoine, et al., (2008)
Acoustic Complexity Index	ACI	A measure of spectrogram complexity based on frequency bins	Pieretti, Farina, & Morri, (2011)

To identify sites with similar acoustic properties, a dendrogram based on Euclidean distance was used and sites were classified based on their acoustic soundscape as described by the variables in Appendix S1. We classified scaled summary statistics of AIs for each site using Ward's method based on the Euclidean distance between site hours. The R function “hclust” was used to minimize the variance between clusters. To identify acoustic variables that best described the variation between sites and hours, we then ran a principal component analysis (PCA) using the R package FactoMineR (Lê, Josse, & Husson, 2008) with summary statistics of each AI as variables and site hours as individual observations (in total 12 sites*24 hr = 288 individual observations).

#### 2.3.2 Spatial and temporal variation in soundscapes

We then analyzed and described sites and hours according to their acoustic properties using summary statistics of AIs and results from the PCA. Coordinates of individual observations along the PCA dimensions were extracted from the PCA and either grouped by sites (24 data points per site) for spatial analysis or hours (12 data points per hour) to analyze temporal patterns. To generate a single coordinate for each site or hour, we calculated the mean and standard deviation of coordinates for each site or hour using the R package “dplyr” (Wickham, François, Henry, & Müller, 2019).

Acoustic differences between groups were described by calculating the mean, standard deviation and minimum of each AI to describe variation, central tendency and outer position in these data. For easier interpretation, we used inverted values of mean and minimum of the H index, as 0 indicates high envelope and spectral complexity and 1 indicates no envelope and spectral complexity.

## 3 RESULTS

### 3.1 Variation in acoustic soundscapes between sites

**FIGURE 1:**
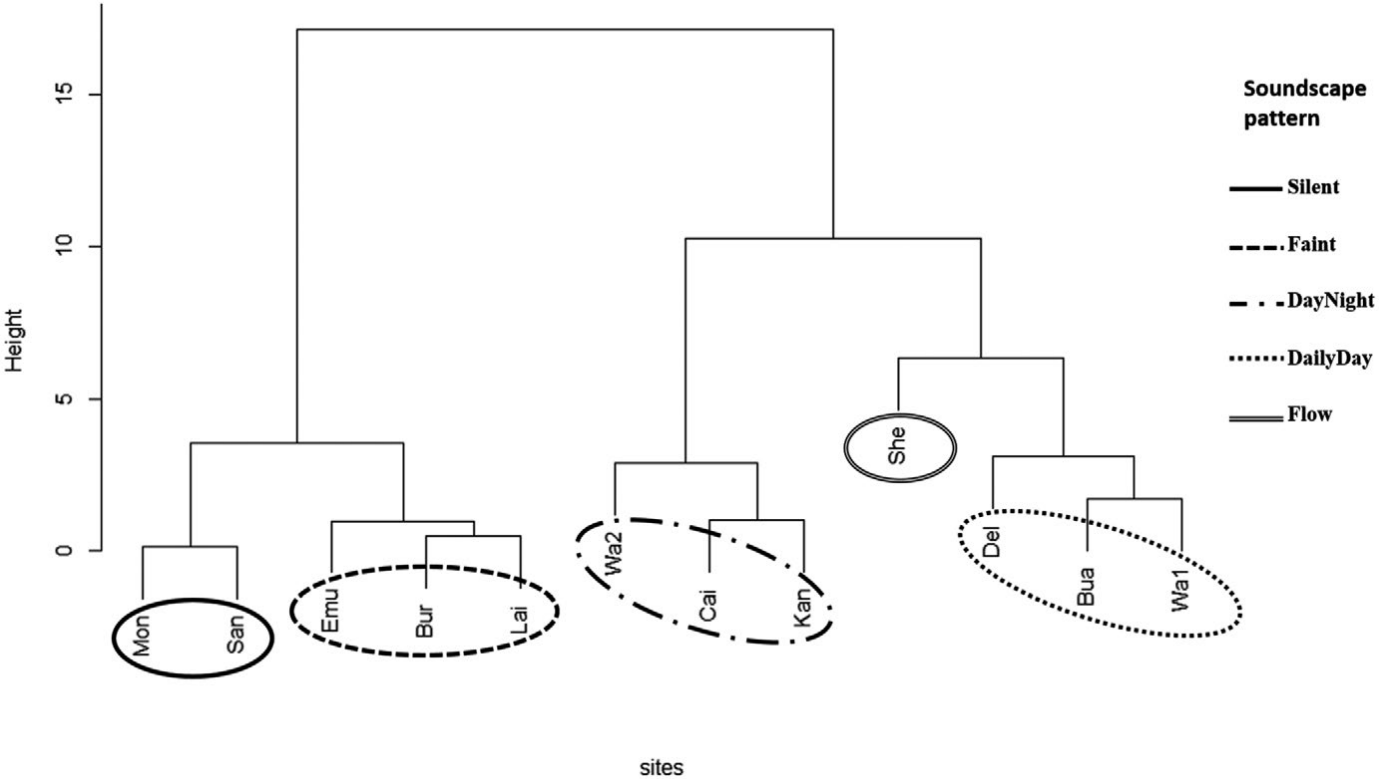
Euclidean dendrogram with five groups. Sites within one circle indicate sites with similar acoustic properties and soundscape pattern. Height represents Euclidean distance between nodes (i.e., the total within‐cluster error sum of squares)

**FIGURE 2:**
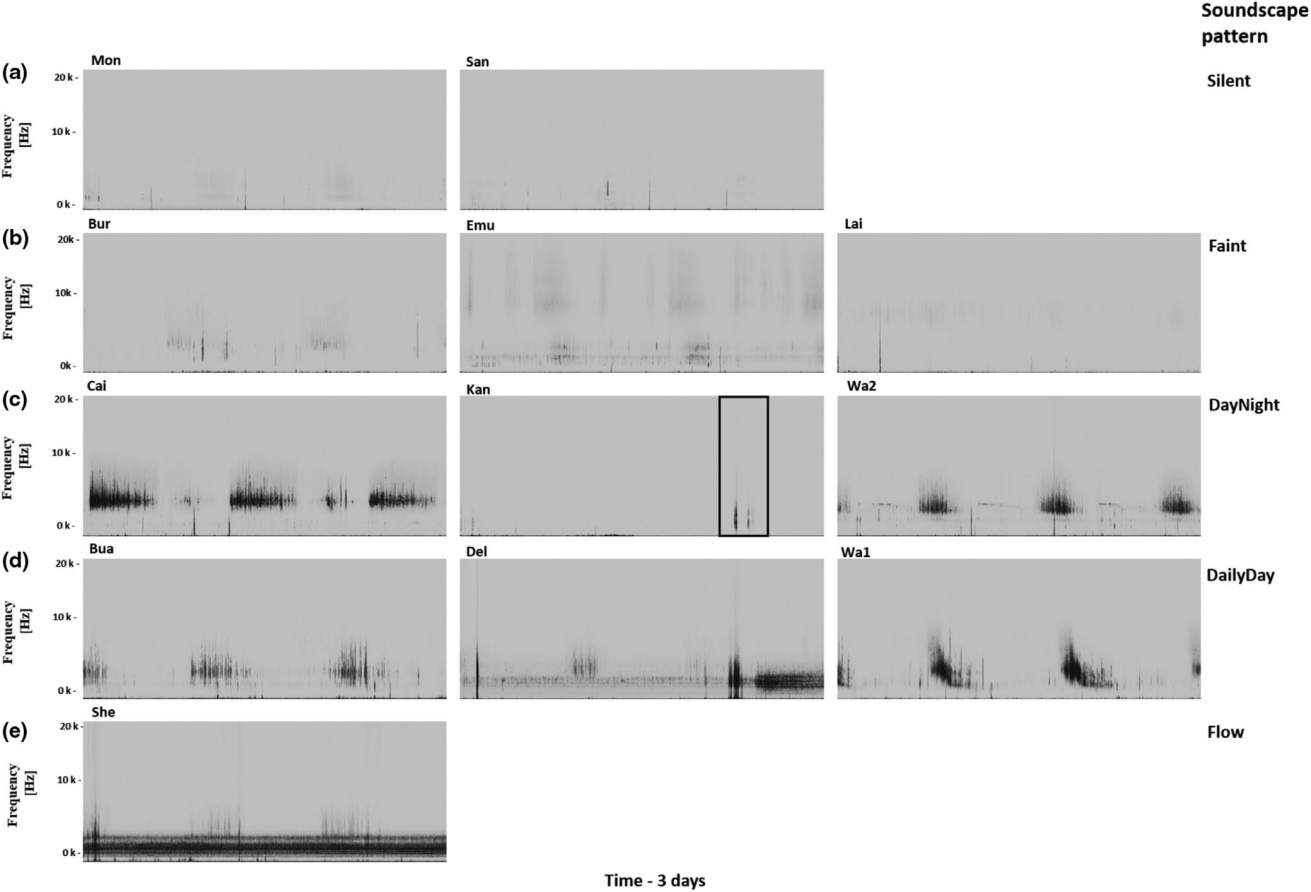
Spectrograms of each site grouped according to their acoustic properties. (a) sites in group “Silent,” (b) sites in group “Faint,” (c) sites in group “DayNight,” (d) sites in group “DailyDay,” (e) group “Flow.” Rectangle in “Kan” site indicates rain. Darker patterns indicate a higher amplitudes

Sites with similar acoustic properties were identified and described using a dendrogram and summary statistics of AIs. Acoustic soundscapes were initially separated into three groups at a height of 10 (Figure 1). Combining visual inspection of spectrograms and dendrogram showed that a separation of sites into 5 groups at height 4 is more appropriate (Figures 1 and 2). Each group was characterized and “named” according to the visual representation of the combined acoustic signal. A “Silent” group was characterized by little to no visual patterns (Figure 2a), while the group denoted “Faint” showed some dim, but nevertheless distinct, patterns within the spectrogram (Figure 2b). Spectrograms of group “DayNight,” except for site “Kan,” exhibited repeated acoustical patterns during day and night with day‐time patterns being darker on the soundscape tracing, indicating higher activity during these hours (Figure 2c). Spectrograms of sites in the “DailyDay” group showed defined dark patterns during the day and no distinct visible signal at night (Figure 2d), while the “Flow” group was characterized by a continuous dark pattern in the lower frequency band (Figure 2e).

**FIGURE 3:**
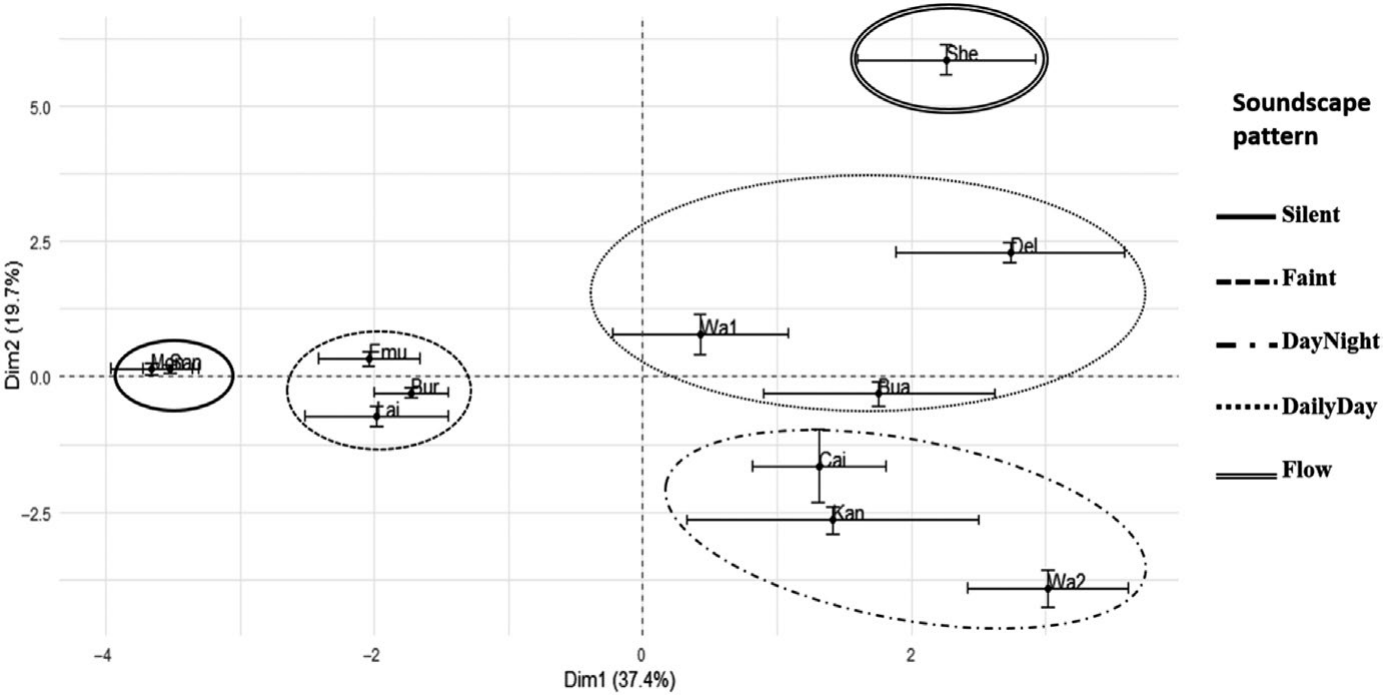
Sites along first two dimensions of PCA. Ellipses indicate sites with similar acoustic properties and soundscape patterns

According to PCA, over 56% of the variation between sites and hours could be explained in two dimensions (Appendix S2) with a distinct distribution of two groups along the first dimension (Figure 3). Variation in the distribution of sites along Dimension 1 was correlated with mean spectral entropy (H_mean_), variation of spectral complexity (ACI_SE.mean_, ACI_CI.mean.0.95_, ACI_std.dev_, ACI_coef.var_), and variation in amplitude envelope (M_std.dev_, *M_SE_*_.mean_, M_CI.mean.0.95_) of hours within a site (Table 3, Appendix S3). Variation between sites along Dimension 2 was correlated with median amplitude envelope, frequency of amplitude envelope and minimum amplitude envelope (M_median_, M_sum_, M_min_) of hours within a site (Table 3, Appendix S3).

**TABLE 3:** Variables contribution (>0.7) to the principal component analysis

Variables that contribute the most to Dimension 1	Value	Variables that contribute the most to Dimension 2	Value
H_mean	−0.75716	M_sum	0.722416
ACI_SE.mean	0.744357	M_min	0.715212
ACI_CI.mean.0.95	0.74435	M_median	0.703503
ACI_std.dev	0.741106		
ACI_coef.var	0.737254		
M_std.dev	0.726835		
M_SE.mean	0.723637		
M_CI.mean.0.95	0.723513		
ACI_max	0.703344		

When the groups identified through the classification were mapped onto the PCA, the group “Silent” was negatively correlated with Dimension 1, “Faint” correlated negatively with both dimensions, “DayNight” correlated positively with Dimension 1 and negatively with Dimension 2 and “DailyDay” and “Flow” correlated positively with both dimensions (Figure 3, Appendix S4).

### 3.2 Acoustic properties of groups

**FIGURE 4:**
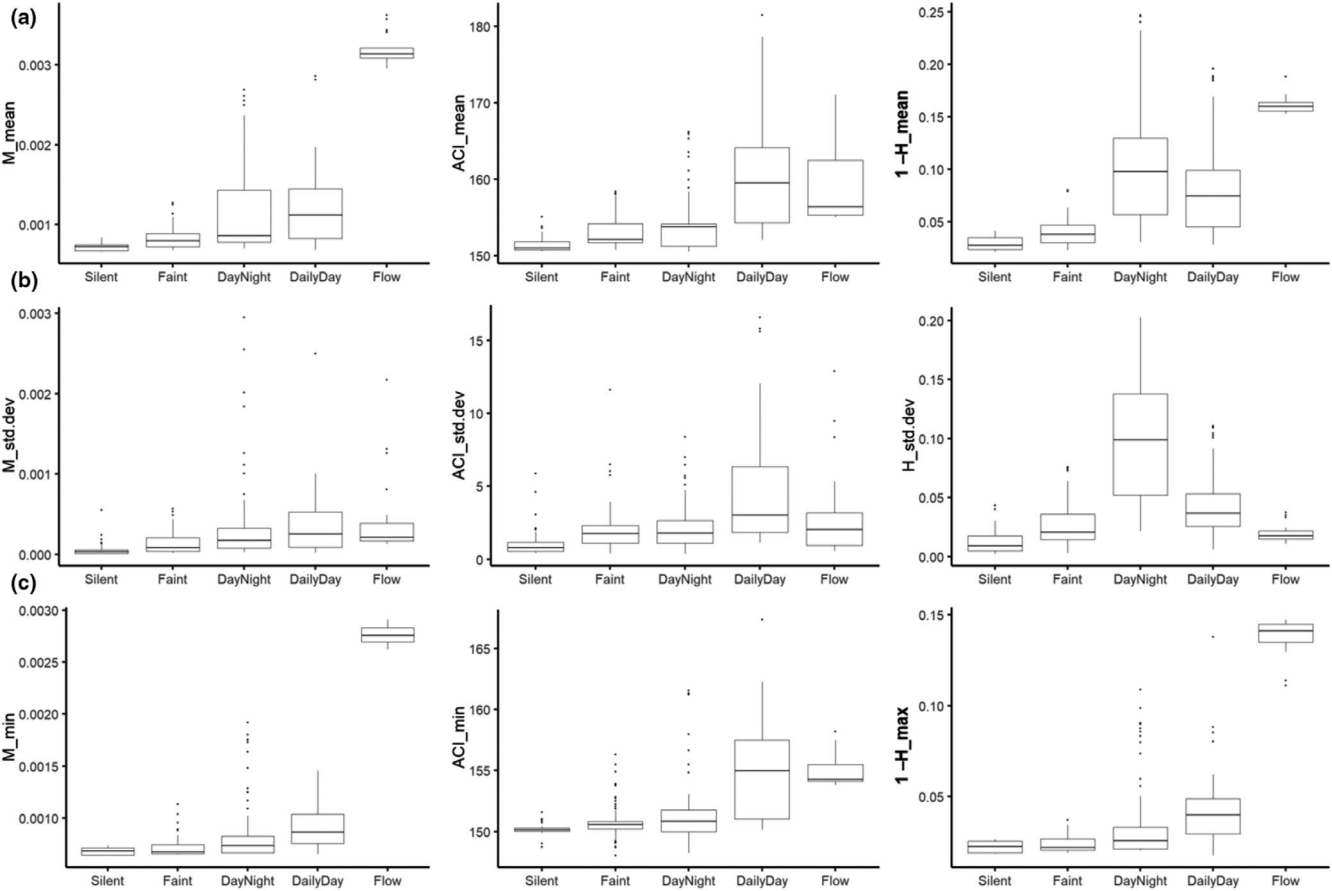
Boxplot with hourly summary statistics of AIs in groups “Silent,” “Faint,” “DayNight,” “DailyDay,” and “Flow”. (a) Average hourly mean, (b) average hourly standard deviation, (c) average hourly minimum. Mean and minimum values for H index are represented inverse for easier comparison

To analyze acoustic properties of each group found through the PCA, we looked at summary statistics of each AI separately. To further explore how summary statistics of AIs describe soundscapes of groups, we analyzed within‐ and between‐group acoustic properties. The group “Silent” had the lowest values and variation for almost all hourly summary statistics of each AI (Figure 4) and no obvious pattern in the spectrograms of sites “Mon” and “San” (Figure 2a). Summary statistics for sites within the group “Faint” were similar to “Silent,” though exhibited higher values and variation for most summary statistics (Figure 4). Sites within “DayNight” displayed high variation within the hourly means of H and M and the standard deviation of H, while hourly statistics of ACI were similar to sites within “Faint” (Figure 4). Spectral inspection revealed different patterns occurring during day and night in the sites “Cai” and “Wa2” of group “DayNight” while only a rain pattern was seen in site “Kan” (Figure 2c). The highest variation of hourly statistics of ACI were from sites within the “DailyDay” group (Figure 4), which exhibited a diurnal sound pattern during daytime (Figure 2d). Sites within the group “Flow” had the highest hourly mean and minimum for M and H and a low variation of hourly standard deviation for both (Figure 4, Table 4).

**TABLE 4:** Visual and statistical description of groups

Group	Visual description	Summary statistics
Silent	No visual pattern in spectrogram	Low values and variation in all summary statistics
Faint	Little visual pattern	Similar to "Silent" but higher and bigger variation of summary statistics values
DayNight	Distinct pattern during day and night times with day time pattern being darker	Big variation of H_mean, M_mean, and H_std.dev; ACI values similar to "Faint"
DailyDay	Pattern visible only during the day	Biggest variation of ACI
Flow	Constant pattern in the lower frequency band	Highest value for M_mean, M_min, H_mean, and H_min

### 3.3 Temporal variation

**FIGURE 5:**
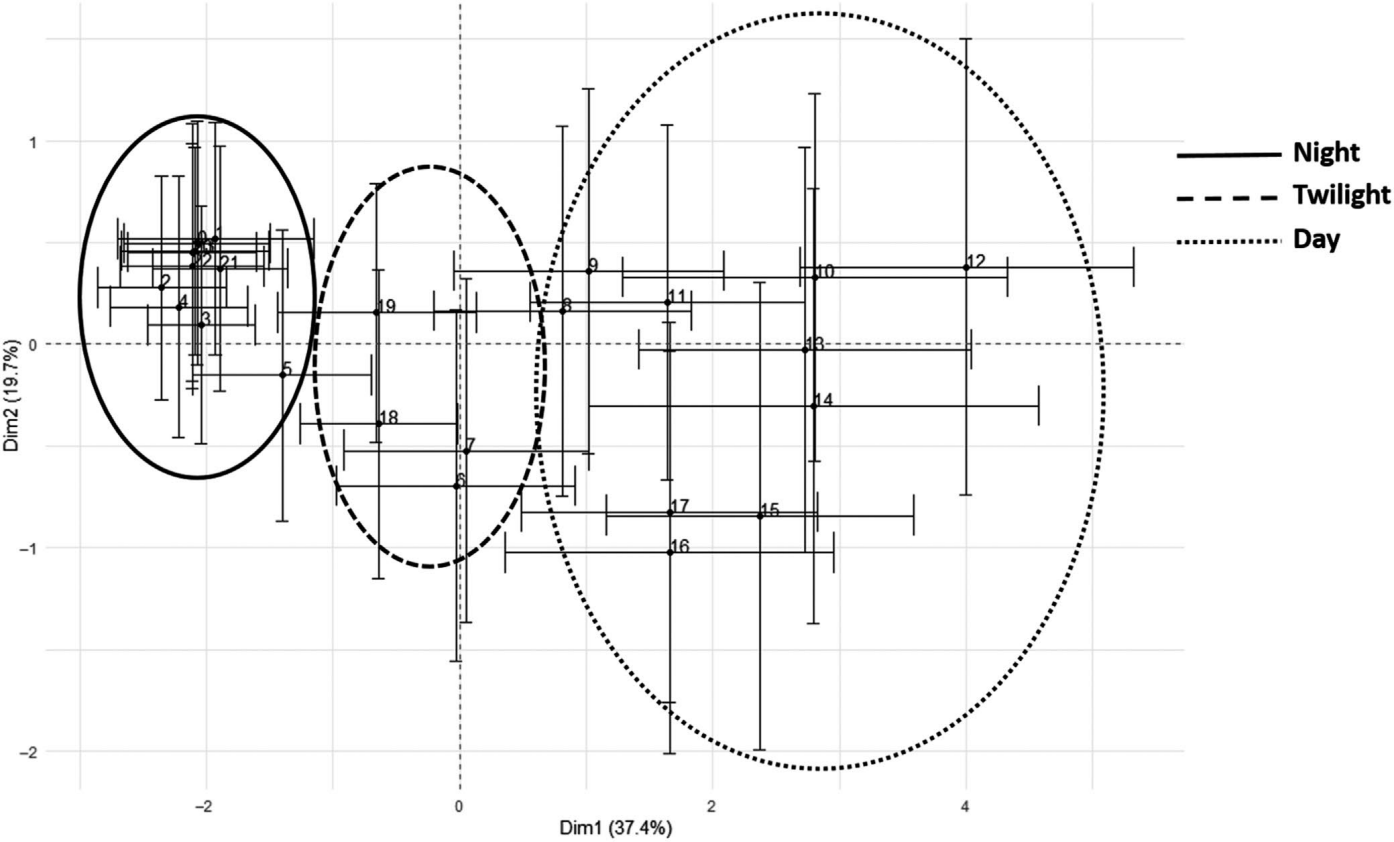
Hours along the first two dimensions of PCA. Ellipses indicate hours within the same daytime

To explore acoustic variation between times of day, we performed a PCA with site hours as individual observations and grouped the results by hours. When plotting site soundscapes on PCA dimensions by the time of day, a clear pattern of temporal change emerged. Night hours (20:00 until 05:00) grouped closely together within the PCA and were negatively correlated with Dimension 1. Twilight hours (06:00, 07:00. 18:00, and 19:00) grouped within the center of the PCA, though the dusk hours were closer to night hours on the negative side of Dimension 1, while dawn hours were positioned closer to daytime hours. Soundscapes recorded during daytime hours (08:00 until 17:00) had the broadest distribution and standard deviation within hours. Earlier hours (08:00 until 12:00) correlated positively with Dimension 2, later hours correlated negatively with Dimension 2 (Figure 5, Appendix S5).

## 4 DISCUSSION

This study represents the first attempt to investigate soundscape diversity of freshwater streams over a broad spatial scale. The aim of the study was to characterize soundscapes across multiple freshwater streams, classify these streams according to their soundscape and explore the use of acoustic indices to describe freshwater stream soundscapes. Our results demonstrate that soundscapes in freshwater streams can be highly variable in both space and time. Even among similar streams in the same region, soundscapes differed greatly. A variety of papers have examined sounds occurring in single freshwater bodies (Gottesman et al., 2020; Linke et al., 2020), but this is the first study to examine variation in soundscapes across 12 streams, of similar size, in the same region. Across these sites, we identified five distinct groups with similar acoustic characteristics as described using summary statistics of AIs. While many studies have used AIs to predict, assess, and monitor biodiversity (Buxton et al., 2018; Gasc, Sueur, Pavoine, Pellens, & Grandcolas, 2013; Jérôme Sueur, Pavoine, Hamerlynck, & Duvail, 2008), summary statistics of AIs have not previously been used to characterize soundscapes in their entirety.

### 4.1 Acoustic properties of groups

Each stream soundscape examined here was unique but also exhibited some acoustic characteristics that were generic across other streams. Acoustic variation within stream soundscapes of sites in the groups “Silent” and “Faint” was smaller compared with that of other groups (indicated by the smaller ellipse in Figure 2). This is also reflected in spectrograms of groups “Faint” and “Silent” which did not show much diversity compared with other groups indicating a quiet and relatively simple acoustic composition. A possible explanation for the lack of sonic activity within these sites could be the absence, or low abundance, of soniferous species and/or sediment movement (Desjonquères et al., 2015), however, this demands further investigation.

Acoustic variation between stream soundscapes within groups “DayNight” and “DailyDay” was the largest compared to soundscape variation within other groups. Summary statistics for both acoustic groups displayed similar values for the acoustic index M indicating a high amplitude in stream soundscapes which aligned with the dark patterns in the spectrogram. Sites in group “DailyDay” had the highest values of summary statistics for acoustic index ACI, indicating complexity of soundscapes. The high standard deviation along the first dimension of the PCA in “DailyDay” sites can be explained by the noncontinuous sound activity over time. During the day, “DailyDay” sites displayed a higher sonic activity than at night leading to a higher variability of ACI in total, meaning soundscapes in these sites were not continuously complex, but rather exhibited diurnal variation.

Group “DayNight” had high summary statistics for acoustic index H indicating greater complexity over time that was also highly variable between hours and sites within this group. The only unusual site in the “DayNight” group was “Kan”. The spectrograms for sites “Cai” and “Wa2” showed a daily day‐night pattern while site “Kan” only exhibited sonic activity during the rain. Acoustic indices have been shown to be biased by the presence of rainfall (Depraetere et al., 2012; Fairbrass, Rennett, Williams, Titheridge, & Jones, 2017; Towsey, Wimmer, Williamson, & Roe, 2014) which would explain the surprising pairing of “Kan” with “Cai” and “Wa2” in the dendrogram. The aim of this study was to investigate the use of acoustic indices without preprocessing acoustic data. In the future, detection and removal of rainfall sounds (Metcalf, Lees, Barlow, Marsden, & Devenish, 2020) could be considered to reduce the influence of external sounds.

Like rainfall, the sound of water flow can display very high amplitudes (Tonolla et al., 2011) and therefore mask other sounds. The soundscape from the Group “Flow,” represented by the single site “She,” was continuously loud and the most complex over time, as indicated by little variation in the summary values for the AIs of M_mean_, high M_minimum_, high 1‐H_mean_, and high 1‐H_maximum_. That said, on examining the spectrogram (Figure 2), we found underlying soundscape patterns that were distinct from flow. This is evidence for the masking effect of flow–also coherent with the findings of Linke et al. (2020) who described the effect of dominant sounds on acoustic indices. Group “Flow” comprised only one site, further investigation of whether our method, that is, summary statistics of AIs would also work with fast‐flowing streams/rivers is needed.

Other methods to describe and compare soundscapes include the use of manual annotation or calculating the acoustic dissimilarity index between a pair of soundscapes. While manual annotation provides a more detailed description of the soundscape (Linke et al., 2020) it is very labor intensive and sometimes only takes specific sound into account (Desjonquères et al., 2015; Gottesman et al., 2020). Studies in the terrestrial realm used dissimilarity indices to compare soundscapes of different environments (Depraetere et al., 2012) or different times of day (Gasc, Sueur, Pavoine, et al., 2013). The use of a dissimilarity index is an effective way to compare soundscapes with each other, it does not characterize them (Sueur et al., 2014). Our aim was not only to compare, but also to characterize soundscapes of different creeks. Our technique facilitates a description of each soundscape individually as well as a comparison of soundscapes between sites, sites within groups and groups with each other.

Here, we have demonstrated that summary metrics of acoustic indices can describe soundscapes in freshwater streams in the same way as biotic indices can describe biological diversity within ecosystems and hydrological indices can describe hydrological diversity in rivers and streams (Kennard et al., 2010; Puckridge, Sheldon, Walker, & Boulton, 1998). We recorded 12 freshwater sites, twice as many as previous studies, and found five distinct sound patterns originating from both biotic and abiotic sources. Studies relating underwater sounds to species and stream condition are still limited (Desjonquères et al., 2015; Gottesman et al., 2020). Therefore, further research is needed to make broader decisions about species abundance and “health” of streams using acoustic indices and associated summary metrics.

### 4.2 Temporal variation of soundscapes in streams

Previous studies have used acoustic indices in freshwater systems to describe temporal acoustic patterns (Gottesman et al., 2020) and temporal frequency‐specific patterns (Linke et al., 2020). Here, our main aim was to use summary statistics of AIs to characterize temporal patterns in soundscapes of different streams. We found that we could also use summary statistics of AIs to describe daytimes across streams as most streams showed diurnal variation in their soundscape. Similar to Gottesman et al. (2020), night‐time hours showed less sonic difference between hours, indicated by small ellipse and less variation between sites, than that of day‐time hours (Figure 5). Interestingly, dusk hours were more acoustically related to night hours, while soundscapes during dawn hours were closer to those of day‐time hours. While Gottesman et al. (2020) recorded very little sonic activity during twilight, many studies in other habitats have identified high acoustic activity at dusk and dawn (Depraetere et al., 2012; Radford, Jeffs, Tindle, & Montgomery, 2008).

Most differences in stream soundscapes identified in our study were observed during the daytime. During daytime, there was significant variation in soundscapes both between hours and among sites. This is contrary to a previous study conducted in Australia in which most biological sound activity occurred during night (Linke et al., 2020). A further separation of early and late day‐time hours along the second dimension indicates earlier hours displaying higher sonic activity than late hours. This is most likely due to different species occurring during different times of day (Gottesman et al., 2020; Linke et al., 2020) or changing their sonic behavior throughout the day (Desjonquères et al., 2020; Rountree & Juanes, 2017). The detection of a clear separation between night, twilight, and day hours further indicates that using summary statistics of AIs can characterize diurnal variation in freshwater streams.

## 5 CONCLUSION

Soundscapes in streams are diverse and unique, although they exhibit similar acoustic patterns across different sites. The technique presented here allows a simple and fast investigation of streams with similar acoustic properties and the ability to characterize them in a holistic and universal way. Further research is needed to understand why soundscapes in freshwater streams differ and how they will change over time. While we developed this technique in freshwater streams it is also applicable to other acoustic realms.

## CONFLICT OF INTEREST

The authors declare that they have no known competing financial interests or personal relationships that could have appeared to influence the work reported in this paper.

## AUTHOR CONTRIBUTION

**Emilia Decker:** Conceptualization (equal); Data curation (lead); Formal analysis (lead); Methodology (equal); Visualization (lead); Writing‐original draft (lead); Writing‐review & editing (lead). **Brett Parker:** Data curation (equal); Investigation (supporting); Methodology (supporting); Visualization (equal); Writing‐review & editing (equal). **Simon Linke:** Conceptualization (equal); Methodology (equal); Supervision (equal); Writing‐review & editing (equal). **Samantha Capon:** Conceptualization (equal); Methodology (supporting); Supervision (equal); Writing‐review & editing (equal). **Fran Sheldon:** Conceptualization (equal); Methodology (equal); Supervision (equal); Writing‐review & editing (equal). 

## Supporting information

Appendix S1

Appendix S2

Appendix S3

Appendix S4

Appendix S5

## Data Availability

Data are available on figshare: 10.6084/m9.figshare.11905536.

## References

[ece36251-bib-0001] Amoser, S. , Wysocki, L. E. , & Ladich, F. (2004). Noise emission during the first powerboat race in an Alpine lake and potential impact on fish communities. The Journal of the Acoustical Society of America, 116(6), 3789–3797. 10.1121/1.1808219 15658729

[ece36251-bib-0002] Anderson, K. A. , Rountree, R. A. , & Juanes, F. (2008). Soniferous Fishes in the Hudson River. Transactions of the American Fisheries Society, 137(2), 616–626. 10.1577/T05-220.1

[ece36251-bib-0003] Belmar, O. , Bruno, D. , Guareschi, S. , Mellado‐Díaz, A. , Millán, A. , & Velasco, J. (2019). Functional responses of aquatic macroinvertebrates to flow regulation are shaped by natural flow intermittence in Mediterranean streams. Freshwater Biology, 64(5), 1064–1077. 10.1111/fwb.13289

[ece36251-bib-0004] Bruno, D. , Gutiérrez‐Cánovas, C. , Velasco, J. , & Sánchez‐Fernández, D. (2016). Functional redundancy as a tool for bioassessment: A test using riparian vegetation. Science of the Total Environment, 566–567, 1268–1276. 10.1016/j.scitotenv.2016.05.186 27277207

[ece36251-bib-0005] Bunn, S. E. , Abal, E. G. , Smith, M. J. , Choy, S. C. , Fellows, C. S. , Harch, B. D. , … Sheldon, F. (2010). Integration of science and monitoring of river ecosystem health to guide investments in catchment protection and rehabilitation. Freshwater Biology, 55(SUPPL. 1), 223–240. 10.1111/j.1365-2427.2009.02375.x

[ece36251-bib-0006] Buxton, R. T. , Agnihotri, S. , Robin, V. V. , Goel, A. , Balakrishnan, R. , State, C. , … Pradesh, A. (2018). Acoustic indices as rapid indicators of avian diversity in different land‐use types in an Indian biodiversity hotspot. Journal of Ecoacoustics, 2(#GWPZVD), 1–17.

[ece36251-bib-0007] Cadenasso, M. L. , Pickett, S. T. A. , & Grove, J. M. (2006). Dimensions of ecosystem complexity: Heterogeneity, connectivity, and history. Ecological Complexity, 3(1), 1–12. 10.1016/j.ecocom.2005.07.002

[ece36251-bib-0008] Clulow, S. , Mahony, M. , Elliott, L. , Humfeld, S. , & Gerhardt, H. C. (2017). Near‐synchronous calling in the hip‐pocket frog *Assa darlingtoni* . Bioacoustics, 26(3), 249–258. 10.1080/09524622.2016.1260054

[ece36251-bib-0009] Department of Infrastructure Local Government and Planning (2017). South East Queensland Regional Plan 2017 Shaping SEQ. Retrieved from https://planning.dilgp.qld.gov.au/planning/better‐planning/state‐planning/seqrp

[ece36251-bib-0010] Depraetere, M. , Pavoine, S. , Jiguet, F. , Gasc, A. , Duvail, S. , & Sueur, J. (2012). Monitoring animal diversity using acoustic indices: Implementation in a temperate woodland. Ecological Indicators, 13(1), 46–54. 10.1016/j.ecolind.2011.05.006

[ece36251-bib-0011] Desjonquères, C. , Rybak, F. , Castella, E. , Llusia, D. , & Sueur, J. (2018). Acoustic communities reflects lateral hydrological connectivity in riverine floodplain similarly to macroinvertebrate communities. Scientific Reports, 8(1), 1–11. 10.1038/s41598-018-31798-4 30258085 PMC6158236

[ece36251-bib-0012] Desjonquères, C. , Rybak, F. , Depraetere, M. , Gasc, A. , Le Viol, I. , Pavoine, S. , & Sueur, J. (2015). First description of underwater acoustic diversity in three temperate ponds. PeerJ, 3, e1393 10.7717/peerj.1393 26587351 PMC4647551

[ece36251-bib-0013] Desjonquères, C. , Rybak, F. , Ulloa, J. S. , Kempf, A. , Bar Hen, A. , & Sueur, J. (2020). Monitoring the acoustic activity of an aquatic insect population in relation to temperature, vegetation and noise. Freshwater Biology, 65, 107–116. 10.1111/fwb.13171

[ece36251-bib-0014] Elemans, C. , Rasmussen, J. H. , Herbst, C. T. , Düring, D. N. , Zollinger, S. A. , Brumm, H. , … Švec, J. G. (2015). Universal mechanisms of sound production and control in birds and mammals. Nature Communications, 6, 10.1038/ncomms9978 PMC4674827 26612008

[ece36251-bib-0015] Fairbrass, A. J. , Rennett, P. , Williams, C. , Titheridge, H. , & Jones, K. E. (2017). Biases of acoustic indices measuring biodiversity in urban areas. Ecological Indicators, 83, 169–177. 10.1016/j.ecolind.2017.07.064

[ece36251-bib-0016] Farina, A. (2014). Soundscape ecology: Principles, patterns, methods and applications. In Soundscape Ecology: Principles, Patterns, Methods and Applications 10.1007/978-94-007-7374-5

[ece36251-bib-0017] Farina, A. , & Fuller, S. (2017). Landscape Patterns and Soundscape Processes In Farina A. & Gage S. H. (Eds.), Ecoacoustics (pp. 193–209). Somerset, NJ: John Wiley and Sons 10.1002/9781119230724.ch11

[ece36251-bib-0018] FFmpeg team , (2019). FFmpeg. Retrieved from http://ffmpeg.org/

[ece36251-bib-0019] Gasc, A. , Sueur, J. , Jiguet, F. , Devictor, V. , Grandcolas, P. , Burrow, C. , … Pavoine, S. (2013). Assessing biodiversity with sound: Do acoustic diversity indices reflect phylogenetic and functional diversities of bird communities? Ecological Indicators, 25, 279–287. 10.1016/j.ecolind.2012.10.009

[ece36251-bib-0020] Gasc, A. , Sueur, J. , Pavoine, S. , Pellens, R. , & Grandcolas, P. (2013). Biodiversity sampling using a global acoustic approach: contrasting sites with Microendemics in New Caledonia. PLoS ONE, 8(5), 1–16. 10.1371/journal.pone.0065311 PMC3667079 23734245

[ece36251-bib-0021] Gottesman, B. L. , Francomano, D. , Zhao, Z. , Bellisario, K. , Ghadiri, M. , Broadhead, T. , … Pijanowski, B. C. (2020). Acoustic monitoring reveals diversity and surprising dynamics in tropical freshwater soundscapes. Freshwater Biology, 65, 117–132. 10.1111/fwb.13096

[ece36251-bib-0022] Grosjean, P. (2018). Package for Analysis of Space‐Time Ecological Series. Retrieved from https://github.com/phgrosjean/pastecs

[ece36251-bib-0023] Heltshe, J. F. , & Forrester, N. E. (1983). Estimating Species Richness Using the Jackknife Procedure. International Biometric Society, 39(1), 1–11. 6871338

[ece36251-bib-0024] Indraswari, K. , Bower, D. S. , Tucker, D. , Schwarzkopf, L. , Towsey, M. , & Roe, P. (2018). Assessing the value of acoustic indices to distinguish species and quantify activity: A case study using frogs. Freshwater Biology, (March, 2019), 65, 1–11. 10.1111/fwb.13222

[ece36251-bib-0025] Izsák, J. , & Papp, L. (2000). A link between ecological diversity indices and measures of biodiversity. Ecological Modelling, 130(1–3), 151–156. 10.1016/S0304-3800(00)00203-9

[ece36251-bib-0026] Junk, W. , Bayley, P. B. , & Sparks, R. E. (1989). The flood pulse concept in river‐floodplain systems. Canadian Special Publication of Fisheries and Aquatic Sciences, 106, 110–127.

[ece36251-bib-0027] Kennard, M. J. , Pusey, B. J. , Olden, J. D. , MacKay, S. J. , Stein, J. L. , & Marsh, N. (2010). Classification of natural flow regimes in Australia to support environmental flow management. Freshwater Biology, 55(1), 171–193. 10.1111/j.1365-2427.2009.02307.x

[ece36251-bib-0028] Laliberte, E. , & Legendre, P. (2010). A distance‐based framework for measuring functional diversity from multiple traits. Ecology, 91(1), 299–305. 10.1890/08-2244.1 20380219

[ece36251-bib-0029] Lê, S. , Josse, J. , & Husson, F. (2008). FactoMineR: An R Package for Multivariate Analysis. Journal of Statistical Software, 25(1), 1–18. 10.1016/j.envint.2008.06.007

[ece36251-bib-0030] Leopold, L. B. , & Maddock, T. J. (1953). The Hydraulic Geomtry of Stream Channels and Some Physiographic Implications. In Geological Survey Professional Paper 252.

[ece36251-bib-0031] Linke, S. , Decker, E. , Gifford, T. , & Desjonquères, C. (2020). Diurnal variation in freshwater ecoacoustics: Implications for site‐level sampling design. Freshwater Biology, 65, 86–95. 10.1111/fwb.13227

[ece36251-bib-0032] Madsen, P. T. (2005). Marine mammals and noise: Problems with root mean square sound pressure levels for transients. Acoustical Society of America, 117(6), 3952–3957. Retrieved from http://marinebioacoustics.com/files/2005/Madsen_2005.pdf 10.1121/1.1921508 16018497

[ece36251-bib-0033] Metcalf, O. C. , Lees, A. C. , Barlow, J. , Marsden, S. J. , & Devenish, C. (2020). hardRain: An R package for quick, automated rainfall detection in ecoacoustic datasets using a threshold‐based approach. Ecological Indicators, 109, 105793 10.1016/j.ecolind.2019.105793

[ece36251-bib-0034] Montie, E. W. , Vega, S. , & Powell, M. (2015). Seasonal and spatial patterns of fish sound production in the May River, South Carolina. Transactions of the American Fisheries Society, 144(4), 705–716. 10.1080/00028487.2015.1037014

[ece36251-bib-0035] Montogomery, D. R. , & Buffington, J. M. (1997). Channel‐reach morpohology in mountain drainage basins. Geological Society of America Bulletin, 109(5), 596–612. Retrieved from https://www.uvm.edu/~wbowden/Teaching/Stream_Geomorph_Assess/Resources/Private/Documents/1997_montgomery_buffington_channel_evolution.pdf

[ece36251-bib-0036] Mouillot, D. , Graham, N. A. J. , Villéger, S. , Mason, N. W. H. , & Bellwood, D. R. (2013). A functional approach reveals community responses to disturbances. Trends in Ecology and Evolution, 28(3), 167–177. 10.1016/j.tree.2012.10.004 23141923

[ece36251-bib-0037] Payne, S. R. , Davies, W. J. , & Adams, M. (2009). Research into the practical and policy applications of soundscape concepts and techniques in urban areas (NANR 200). In Defra. Retrieved from. http://usir.salford.ac.uk/27343/

[ece36251-bib-0038] Pieretti, N. , Farina, A. , & Morri, D. (2011). A new methodology to infer the singing activity of an avian community: The Acoustic Complexity Index (ACI). Ecological Indicators, 11(3), 868–873. 10.1016/j.ecolind.2010.11.005

[ece36251-bib-0039] Puckridge, J. T. , Sheldon, F. , Walker, K. F. , & Boulton, A. J. (1998). Flow variability and the ecology of large rivers. Marine and Freshwater Research, 49, 55–72.

[ece36251-bib-0040] Putland, R. L. , Constantine, R. , & Radford, C. A. (2017). Exploring spatial and temporal trends in the soundscape of an ecologically significant embayment. Scientific Reports, 7(1), 1–12. 10.1038/s41598-017-06347-0 28720760 PMC5516011

[ece36251-bib-0041] Radford, C. A. , Jeffs, A. G. , Tindle, C. T. , & Montgomery, J. C. (2008). Temporal patterns in ambient noise of biological origin from a shallow water temperate reef. Oecologia, 156(4), 921–929. 10.1007/s00442-008-1041-y 18461369

[ece36251-bib-0042] Rountree, R. A. , & Juanes, F. (2017). Potential of passive acoustic recording for monitoring invasive species: Freshwater drum invasion of the Hudson River via the New York canal system. Biological Invasions, 19(7), 2075–2088. 10.1007/s10530-017-1419-z

[ece36251-bib-0043] Rountree, R. A. , & Juanes, F. (2018). Potential for use of passive acoustic monitoring of piranhas in the Pacaya‐Samiria National Reserve in Peru. Freshwater Biology, (March, 2018), 65, 55–65. 10.1111/fwb.13185

[ece36251-bib-0044] Sheldon, F. , Erin, E. P. , Boone, E. L. , Sippel, S. , Bunn, S. E. , & Harch, B. D. (2012). Identifying the spatial scale of land use that most strongly influences overall river ecosystem health score. Ecological Applications, 22(8), 2188–2203. 10.1890/11-1792.1 23387119

[ece36251-bib-0045] Soria, M. , Gutiérrez‐Cánovas, C. , Bonada, N. , Acosta, R. , Rodríguez‐Lozano, P. , Fortuño, P. , … Cid, N. (2019). Natural disturbances can produce misleading bioassessment results: Identifying metrics to detect anthropogenic impacts in intermittent rivers. Journal of Applied Ecology, 65, 1–13. 10.1111/1365-2664.13538

[ece36251-bib-0046] Sueur, J. , Aubin, T. , & Simonis, C. (2008). Seewave, a free modular tool for sound analysis and synthesis. Bioacoustics, 18(2), 213–226. 10.1080/09524622.2008.9753600

[ece36251-bib-0047] Sueur, J. , Farina, A. , Gasc, A. , Pieretti, N. , & Pavoine, S. (2014). Acoustic indices for biodiversity assessment and landscape investigation. Acta Acustica United with Acustica, 100(4), 772–781. 10.3813/AAA.918757

[ece36251-bib-0048] Sueur, J. , Pavoine, S. , Hamerlynck, O. , & Duvail, S. (2008). Rapid acoustic survey for biodiversity appraisal. PLoS ONE, 3(12), 10.1371/journal.pone.0004065 PMC2605254 19115006

[ece36251-bib-0049] Sweger, A. L. , & Uetz, G. W. (2016). Characterizing the vibratory and acoustic signals of the “purring” wolf spider, *Gladicosa gulosa* (Araneae: Lycosidae). Bioacoustics, 4622, 1–11. 10.1080/09524622.2016.1160328

[ece36251-bib-0050] Tonolla, D. , Acuña, V. , Lorang, M. S. , Heutschi, K. , & Tockner, K. (2010). A field‐based investigation to examine underwater soundscapes of five common river habitats. Hydrological Processes, 24(22), 3146–3156. 10.1002/hyp.7730

[ece36251-bib-0051] Tonolla, D. , Lorang, M. S. , Heutschi, K. , Gotschalk, C. C. , & Tockner, K. (2011). Characterization of spatial heterogeneity in underwater soundscapes at the river segment scale. Limnology and Oceanography, 56(6), 2319–2333. 10.4319/lo.2011.56.6.2319

[ece36251-bib-0052] Towsey, M. , Wimmer, J. , Williamson, I. , & Roe, P. (2014). The use of acoustic indices to determine avian species richness in audio‐recordings of the environment. Ecological Informatics, 21(100), 110–119. 10.1016/j.ecoinf.2013.11.007

[ece36251-bib-0053] Ulgiati, S. , & Brown, M. T. (2009). Emergy and ecosystem complexity. Communications in Nonlinear Science and Numerical Simulation, 14(1), 310–321. 10.1016/j.cnsns.2007.05.028

[ece36251-bib-0054] Vannote, R. L. , Minshall, G. W. , Cummins, K. W. , Sedell, J. R. , & Cushing, C. E. (1980). The river continuum concept. Canadian Journal of Fisheries and Aquatic Sciences, 37(1), 130–137. 10.1139/f80-017

[ece36251-bib-0055] Villanueva‐Rivera, L. J. , & Pijanowski, B. C. (2016). Package “soundecology”. doi: 10.1016/j.ecolind.2010.11.005.

[ece36251-bib-0056] Ward, J. V. , Tockner, K. , Uehlinger, U. , & Malard, F. (2001). Understanding natural patterns and processes in river corridors as the basis for effective river restoration. Regulated Rivers: Research & Management, 17(4–5), 311–323. 10.1002/rrr.646

[ece36251-bib-0057] Waterways, H. (2018). Healthy land and water. Retrieved from http://hlw.org.au/.

[ece36251-bib-0058] Wickham, H. , François, R. , Henry, L. , & Müller, K. (2019). A Grammar of Data Manipulation. Retrieved from, https://cran.r‐project.org/web/packages/dplyr/dplyr.pdf

[ece36251-bib-0059] Wohl, E. , & Merritt, D. M. (2008). Reach‐scale channel geometry of mountain streams. Geomorphology, 93(3–4), 168–185. 10.1016/j.geomorph.2007.02.014

[ece36251-bib-0060] Wysocki, L. E. , Amoser, S. , & Ladich, F. (2007). Diversity in ambient noise in European freshwater habitats: Noise levels, spectral profiles, and impact on fishes. The Journal of the Acoustical Society of America, 121(5), 2559–2566. 10.1121/1.2713661 17550155

[ece36251-bib-0061] Wysocki, L. E. , Dittami, J. P. , & Ladich, F. (2006). Ship noise and cortisol secretion in European freshwater fishes. Biological Conservation, 128(5), 501–508. 10.1016/j.biocon.2005.10.020

[ece36251-bib-0062] Young, B. A. (2003). Snake bioacoustics: Toward a richer understanding of the behavioral ecology of snakes. The Quarterly Review of Biology, 78(3), 303–325. 10.1086/377052 14528622

[ece36251-bib-0063] Zhou, X. , Li, J. , Tao Shen, H. , Kitsuregawa, M. , & Zhang, Y. (2006). Frontiers of WWW Research and Development – APWeb 2006 (X. Zhou, L. J, H. T. Shen, M. Kitsuregawa, & Y. Zhang, Eds.). Springer, Berlin, Heidelberg.

